# Genetic analysis and prenatal diagnosis of recessive dystrophic epidermolysis bullosa caused by compound heterozygous variants of the *COL7A1* gene in a Chinese family

**DOI:** 10.3389/fped.2022.941201

**Published:** 2022-11-07

**Authors:** Yu Wang, Zhen Song, Lihua Zhang, Na Li, Jie Zhao, Ruifang Yang, Shuhua Ji, Ping Sun

**Affiliations:** ^1^ Prenatal Diagnostic Center of Obstetrics and Gynecology Department, Qilu Hospital of Shandong University, Jinan, China; ^2^ Department of Dermatology, Qilu Hospital of Shandong University, Jinan, China; ^3^ Yinfeng Gene Technology Co. Ltd., Jinan, China

**Keywords:** autosomal recessive dystrophic epidermolysis bullosa, *COL7A1*, high-throughput sequencing analysis, compound heterozygous variants, prenatal diagnosis

## Abstract

**Background:**

Dystrophic epidermolysis bullosa (DEB) is an incurable and inherited skin disorder mainly caused by mutations in the gene encoding type VII collagen (COL7A1). The purpose of this study was to identify the causative genetic variants and further perform genetic diagnosis in a Chinese family affected by DEB.

**Methods:**

High-throughput sequencing was performed to analyze the genetic skin disorder-related genes of parents of the proband, and the variants were further confirmed in the other members by Sanger sequencing. Sanger sequencing, karyotype analysis, and chromosomal microarray analysis (CMA) were used together for prenatal diagnosis after the second pregnancy. The phenotype of the fetus was tracked after the diagnosis and induction of labor. Moreover, skin and muscle pathological examination and whole-exome sequencing (WES) of the skin and muscle tissue of the induced fetus were performed.

**Results:**

Here, we determined two heterozygous variants of the *COL7A1* gene that contributed to the autosomal recessive DEB (RDEB) in the family, i.e., a novel pathogenic variant (c.8335G > T, p.E2779*) and a likely pathogenic variant (c.7957G > A, p.G2653R). Sanger sequencing of amniotic fluid cells showed that the fetus carried the above two compound heterozygous variants, and the karyotype analysis and CMA results showed no abnormality. The clinical phenotype and pathological results of the induced fetus were consistent with the characteristics of DEB. Further, WES analysis also confirmed a novel compound heterozygous variation in COL7A1, consisting of two variants, namely, c.8335G > T and c.7957G > A in the fetus.

**Conclusion:**

This study expands the spectrum of disease-causing variants of *COL7A1* and provides a theoretical basis for diagnosis, genetic counseling, and prognosis of families affected by RDEB

## Introduction

Dystrophic epidermolysis bullosa (DEB) is a common subtype of epidermolysis bullosa characterized by increased skin fragility, tension bullae, atrophic scars, and severe birth defects (1). This disorder comes in two forms based on the pattern of inheritance: autosomal dominant DEB (DDEB; OMIM: 131750) and autosomal recessive DEB (RDEB; OMIM: 226600). The latter form is more severe; RDEB patients typically present with systemic blistering/scarring of the skin and mucous membranes, as well as defects in other organs (2–4).

*COL7A1* is the only well-known disease-causing gene for RDEB, which is located at 3p21.31 and encodes type VII collagen. *COL7A1* is a key component of the anchor fiber of the basement membrane of the skin (5, 6). Anchor fibers can maintain the close connection between the epidermis and the dermis, damage or loss of which could lead to the formation of dense subplate blisters. A total of 997 variants of the *COL7A1* gene have been documented in the LOVD database (https://databases.lovd.nl/shared/genes/COL7A1), and 555 individuals with *COL7A1* variants have been reported.

Prenatal diagnosis of affected family members based on molecular genetic analysis is an important means to prevent the birth of DEB children (7). Here, we studied a Chinese couple who had previously had a daughter born with DEB and were expecting a second child. An abnormal ultrasound suggested that the fetus had features of DEB as well and indicated probable recessive inheritance. Molecular genetic analysis of the parents revealed that each carried a heterozygous variant of *COL7A1* (c.8335G > T and c.7957G>), the gene associated with RDEB. We provided a prenatal diagnosis and genetic counseling based on these findings and ultimately confirmed the clinical and molecular diagnosis of the fetus after the pregnancy was terminated.

## Methods

### Ethical approval and informed consent

This study was approved by the ethics committee of Qilu Hospital of Shandong University (KYLL-202107-075). Written informed consent was obtained for all participants in accordance with the Declaration of Helsinki.

### Clinical report

The proband was a Chinese girl who was the first child of an unrelated healthy couple. Unfortunately, she died of sepsis 7 days after birth. The proband was born with significant skin defects of both legs and feet with bilateral foot varus, and no obvious abnormalities were found in the rest of the skin. She was clinically diagnosed with epidermolysis bullosa, and no related genetic analysis was carried out. The proband's mother got pregnant again, and an ultrasound showed that the fetal feet were abnormal (toe dorsiflexion). A skin disorder-related gene sequencing analysis was performed on the proband's parents to identify the pathogenic mutations. Then, the prenatal diagnosis was carried out. After the fetus was diagnosed genetically, the pregnancy was terminated, and we tracked the clinical phenotype of the induced fetus. We also preserved the skin and muscle tissue samples of the induced fetus for follow-up research.

### Gene panel sequencing

A skin disorder-related gene sequencing analysis comprising a total of 204 genes was performed on the proband's parents to identify the pathogenic mutations. In total, 2 ml of peripheral blood of the proband's parents was drawn, genomic DNA was extracted by the QIAamp DNA Extraction Kit (Qiagen, Germany), and its concentration was determined. The DNA fragments were randomly interrupted for ligation, PCR amplification, and purification and then hybridized with an IDT XGen exome probe (IDT, Lowa, USA) to construct a DNA library. After that, a Novaseq 6000 (Illumina Inc., San Diego, USA) was used for sequencing. We then identified the possible skin disorder-related gene mutations.

### Whole-exome sequencing

Genomic DNA extracted from skin and muscle tissue samples of the induced fetus was fragmented to an average size of 180–280 bp and used to create a DNA library following established Illumina paired-end protocols. The Agilent SureSelect Human All ExonV6 Kit (Agilent Technologies, Santa Clara, CA, USA) was used for exome capture according to the manufacturer's instructions. The Illumina Novaseq 6000 platform (Illumina Inc., San Diego, CA, USA) was used for genomic DNA sequencing by Yinfeng Gene Technology Co., Ltd. (Jinan, China) to generate 150-bp paired-end reads with a minimum coverage of 10× for −97% of the target sequence (mean coverage of 100×).

### Sequence alignment and analysis

After sequencing, base-call file conversion and demultiplexing were performed with bcl2fastq software (Illumina). All data were aligned to the reference sequence (UCSC hg19) using the BWA algorithm. Annotation was performed using ANNOVAR (June 8, 2017). Annotations included minor allele frequencies from the public control data sets and deleteriousness and conservation scores, enabling further filtering and assessing the likely pathogenicity of the variants. Variants were filtered against the public databases (ESP6500 and the 1000 GENOMES Project), the frequency of more than 5% was filtered, and the variants reported as pathogenic or likely pathogenic in the HGMD and ClinVar databases were retained. The pathogenicity of the variants was predicted using online bioinformatics tools (SIFT, PolyPhen2_HDIV, LRT, Mutation Taster, Mutation Assessor, FATHMM, and REVEL). In addition, candidate causative variants were identified according to the function, variation, and genetic pattern of the gene, which were further assessed according to the American College of Medical Genetics (ACMG) guidelines (8).

### Sanger sequencing

To verify the results of high-throughput sequencing analysis, Sanger sequencing of the family members was conducted. Briefly, Primer premier 5.0 software was used to design primers for the exon coding region of the target gene. The 2× PCR MasterMix polymerase (Tiangen, Beijing, China) was used for PCR amplification on the PCR instrument (ABI9700, Life Technology, USA). The PCR products were then directly sequenced on an ABI3500 genetic analyzer (Life Technology). The primers used in this study are listed in Table 1.

**Table 1:** Primers of *COL7A1* used in this study.

ID	Sequence
*COL7A1*-Exon107-7957F	5′-TCAGCCCGTGTCTGAACTC-3′
*COL7A1*-Exon107-7957R	5′-GCCCCATCCTAAGTCCTCAC-3′
*COL7A1*-Exon112-8335F	5′-GTGCTGGGTGAGGGAGGTAG-3′
*COL7A1*-Exon112-8335R	5′-TCCAGAGCTGAGGGAGGTC-3′

### Prenatal diagnosis

Amniocentesis was performed to extract amniotic fluid, and the mutations of the *COL7A1* gene were then verified by Sanger sequencing. Standard karyotype analysis and chromosomal microarray (CMA) testing (Affymetrix CytoScan 750k) were also performed to rule out chromosomal disorders.

### Hematoxylin–eosin (HE) staining

The paraffin sections of the skin and muscle tissue of the aborted fetus were deparaffinized in xylene and then dehydrated with different concentrations of ethanol. The sections were stained in hematoxylin staining solution for 3–8 min, washed with water, and differentiated in 1% acetic acid for a few seconds. Subsequently, the sections were dyed with eosin for 1–3 min, dehydrated, sealed, and observed under a microscope.

## Results

### Clinical presentation and family history

**Figure 1:**
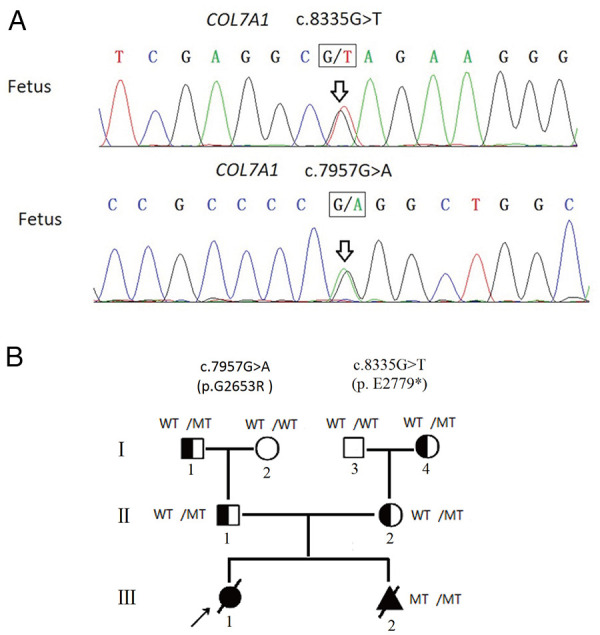
(**A**) Fetus carried two compound heterozygous variants (c.8335G > T, c.7957G > A), which were inherited from the father and the mother separately. (**B**) Pedigree analyses to identify the family members with RDEB. The proband is indicated by an arrow. The black circle indicates affected family members; the triangle represents the induced fetus; MT indicates the heterozygous c.7957G > A variant or heterozygous c.8335G > T variant; diagonal bars through the symbols denote deceased individuals. The proband did not undergo genetic testing.

A Chinese couple came to medical attention after an abnormal ultrasound during their second pregnancy. They had previously had a daughter who was born with skin defects of both legs and feet with bilateral foot varus. These features were considered consistent with DEB, although no genetic testing was performed, and the daughter died of sepsis at 7 days of life. The ultrasound of the second pregnancy revealed similar foot deformity (toe dorsiflexion). The proband's parents were subjected to high-throughput sequencing of skin disorder-related genes. The results showed that the proband's mother carried a novel heterozygous variant of *COL7A1* (c.8335G > T, p.E2779*, NM_000094.4) in exon 112. Further analysis revealed that c.8335G > T, which has not been reported in the previous literature nor the ClinVar database, was inherited from her mother (I-4, Figure 1B; Supplementary Figure S1). The proband's father also carried a heterozygous variant of the *COL7A1* (c.7957G > A, p.G2653R, NM_000094.4) gene in exon 107. The c.7957G > A variant, which has been documented in the ClinVar database and identified in RDEB patients (9, 10), was inherited from his father (I-1, Figure 1B; Supplementary Figure S2).

With the use of the standards and guidelines for the interpretation of sequence variants by ACMG, the evaluation of pathogenicity of the c.8335G > T variant was pathogenic (PVS1 + PM2 + PP4), and the c.7957G > A variant was classified as likely pathogenic (PM1 + PM2 + PM3 + PP3 + PP4). Therefore, we strongly suspected that the proband may have inherited the *COL7A1* gene mutation from her parents, resulting in RDEB.

### Prenatal diagnosis

Amniocentesis was performed at mid-gestation, considering that karyotype and CMA were performed and negative (Supplementary Figure S3), but Sanger sequencing identified both variants of *COL7A1* (Figure 1A). After prenatal diagnosis and genetic counseling, the couple elected to terminate the new pregnancy. Further clinical phenotyping of the induced fetus was performed, and the skin and muscle samples of the induced fetus were preserved for follow-up studies.

### Clinical phenotype of the induced fetus

**Figure 2:**
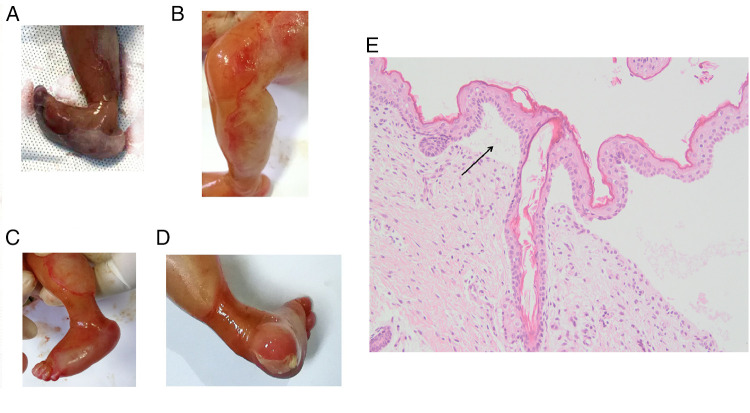
Clinical photographs and HE staining results of an epidermis blister from the affected fetus in the family. (**A**) Pictures taken immediately after induction of labor. (**B–D**) Photos taken after 12 h of refrigeration after induction of labor. Toe dorsiflexion was relieved after refrigeration. (**E**) HE staining picture of the epidermis blister. The magnification is 200 times.

The phenotypes of the induced fetus (male) are as follows: large areas of the epidermis of both legs were missing and the surface was eroded, the shape of both feet was unnatural, the ankles had constricted loops, and the foot epidermis had the appearance of sock-like exfoliation (Figures 2A–D). The clinical characteristics of the induced fetus were highly consistent with DEB and the same as those of the proband. The HE staining results showed the formation of subepidermal blisters (as shown by the arrow in Figure 2E), and a small number of fibroblasts and lymphocytes infiltrated the superficial dermis, which was consistent with the histological changes of DEB (Figure 2E). HE staining is not sufficient to confirm the type of DEB but only to prove the existence of DEB. Therefore, further immunohistochemical and/or genetic analysis is needed to confirm the type of DEB. Subsequently, the clinical phenotype of the induced fetus was consistent with the genetic diagnosis results, further confirming that the fetus was affected with RDEB. Additionally, the variant c.7957G > A was further classified as pathogenic (PM1 + PM2 + PM3_Strong + PP3 + PP4).

### WES analysis of the induced fetus

We performed WES analysis of fetal tissue DNA to rule out other potential causes of the clinical features. WES analysis of the fetal tissue confirmed that there were no other pathogenic variants and also approved a novel compound heterozygous variation in *COL7A1*, consisting of two variants, namely, c.8335G > T and c.7957G > A in the fetus.

## Discussion

EB is a group of rare hereditary skin diseases with high clinical heterogeneity. Due to the complexity of the classification and the number of clinical subtypes of EB, a precise classification of EB subtypes based on clinical presentation alone is challenging. In the present study, since the proband (the only patient) in this family had died at the time of treatment, we only performed genetic testing on her immediate relatives to find the genetic cause. Herein, our data demonstrated that the couple carried a pathogenic variant of *COL7A1* separately, which may be a genetic factor of the affected proband.

Given the severity of DEB, female members of the family with pathogenic variants should perform chorionic villus sampling in early pregnancy to achieve the purpose of early diagnosis and early intervention. For the family presented here, the pregnancy was too far for chorionic villus sampling when the couple came to medical attention, so fetal DNA was obtained *via* amniocentesis. We confirmed that the fetus carried the two compound heterozygous variants of the *COL7A1* gene inherited from its mother and father, separately. After the termination of this pregnancy, we tracked the clinical phenotype of the induced fetus and confirmed that it was consistent with the clinical phenotype of DEB, which further supported the genetic diagnosis. The WES analysis further confirmed that the compound heterozygous variants of the *COL7A1* gene were the only genetic cause of the affected fetus.

DEB can be inherited in an autosomal dominant and autosomal recessive fashion, and the expression of *COL7A1* has been inversely correlated with disease severity (11). There are 14 clinical subtypes of DEB (12), involving more than 200 pathogenic and likely pathogenic variants, among which missense, frameshift, nonsense, and splicing mutations are the four most important mutation types (http://simple-clinvar.broadinstitute.org/). The pattern of inheritance of DEB is usually related to the location and type of *COL7A1* gene mutations. For DDEB, missense mutations are the main cause, and the clinical symptoms of patients are usually mild. In addition to some missense mutations in specific domains, RDEB is generally caused by nonsense, splicing, and frameshift mutations, which can lead to the serious reduction or even absence of the protein expression (2, 13). The clinical symptoms of RDEB patients are generally severe with poor prognosis; even large skin defects are found in some patients (14, 15). Herein, our data revealed that both the father and mother carried a different heterozygous variant in the *COL7A1* gene inherited from one of their parents, but none of these carriers had a DEB-related clinical phenotype. One of them was a nonsense mutation of the *COL7A1* gene, combined with the characteristics of early onset and severe symptoms of the proband; DEB was in line with the autosomal recessive genetic pattern in this family. It is speculated that the proband may carry both the c.8335G > T and c.7957G > A variants, which are in the trans position, forming two compound heterozygote variants of *COL7A1* in the proband, leading to the occurrence of RDEB.

The *COL7A1* spans 118 exons; the c.8335G > T variant identified in this family was in exon 112, leading to a premature termination codon (p.E2779*), resulting in the formation of a truncated *COL7A1* protein. To date, this variant has not been included in public disease-related databases and control and general population databases. As a novel variant, c.8335G > T identified in this study expands the *COLA71* variant-disease spectrum.

Another mutation in the family reported in this study, c.7957G > A in exon 107, is a missense mutation predicted to result in the substitution of glycine by arginine (p.G2653R). Christiano et al. found that a DEB child carried two heterozygous pathogenic variants (c.7411C > T and c.7957G > A) inherited from his parents, respectively, which was consistent with autosomal recessive inheritance (10). Mariath et al. reported a case of recessively inherited DEB patients with both a c.7957G > A heterozygous variant and a c.8109 + 1G > T heterozygous variant, of which c.8109 + 1G > T was a pathogenic variant (14). However, the parental source of the two variants was not confirmed, and it was still uncertain whether the two mutations were in the trans position. As mentioned above, the c.7957G > A variant occurred in three RDEB patients; the PM3 evidence of the c.7957G > A variant could be upgraded to PM3_Strong according to the standards and guidelines for the interpretation of sequence variants published by ClinGen (8, 16). In addition, according to the recommended standard for Sequence Variant Interpretation (PM2) published on ClinGen in September 2020 (https://www.clinicalgenome.org/working-groups/sequence-variant-interpretation/), PM2 evidence should be downgraded to PM2_Supporting. However, in view of the impact of the downgrading of PM2 on the rating of variation, this recommendation has not been widely promoted due to the different views held by researchers (16). For autosomal recessive diseases, PM2 can be used if the variant is not found in the general population or reference population database or the population frequency is less than or equal to 0.00007, according to ClinGen (17). In the present study, the allele frequency of c.7957G > A was 0.00003190, less than 0.00007, so PM2 was still used. Therefore, the c.7957G > A variant was further classified as pathogenic (PM1 + PM2 + PM3_Strong + PP3 + PP4).

In this Chinese family, the mother had experienced two consecutive pregnancies and childbirth histories with RDEB, which brought a heavy psychological burden to the couple. After consultation, they finally decided to use preimplantation genetic diagnosis to screen embryos to increase the probability of having healthy children.

## Conclusion

In summary, this study determined the pathogenic variants and the genetic pattern of DEB in a Chinese family, demonstrating the c.7957G > A and c.8335G > T variants of *COL7A1* in the members of this family with RDEB. According to the genetic results, we successfully performed prenatal diagnosis on the mother, further confirming the key role of rapid genetic diagnosis in prenatal diagnosis. In addition, our data further supplements the *COL7A1* gene variant database, and the diagnosis of this case may provide suggestions for the clinical diagnosis of this kind of single-gene genetic disease.

## Data Availability

The original contributions presented in the study are publicly available. This data can be found here: GSA: HRA002719.
