# Supplementary figures and images for: Genetic analysis and prenatal diagnosis of recessive dystrophic epidermolysis bullosa caused by compound heterozygous variants of the COL7A1 gene in a Chinese family
Source: Front Pediatr. 2022 Nov 7;10:941201. doi: 10.3389/fped.2022.941201 (PMC9676484; doi:10.3389/fped.2022.941201)

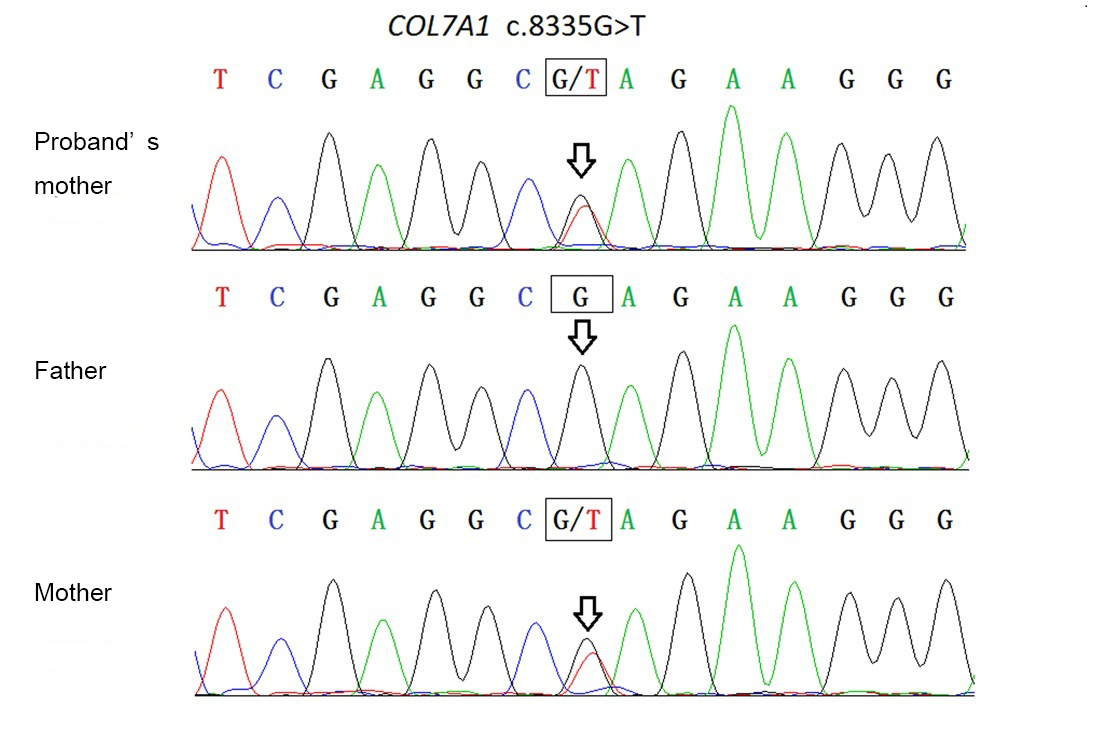

Supplement: Supplementary file 1 [file Image1.tif]

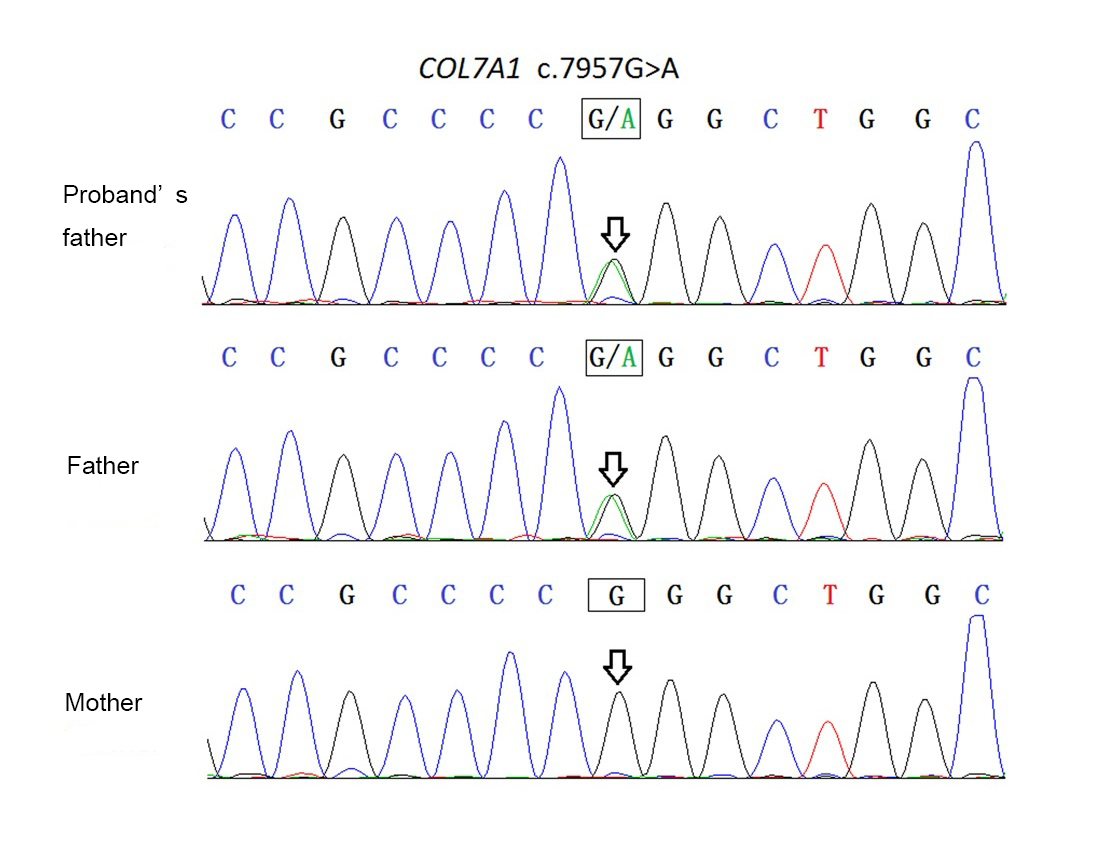

Supplement: Supplementary file 2 [file Image2.tif]

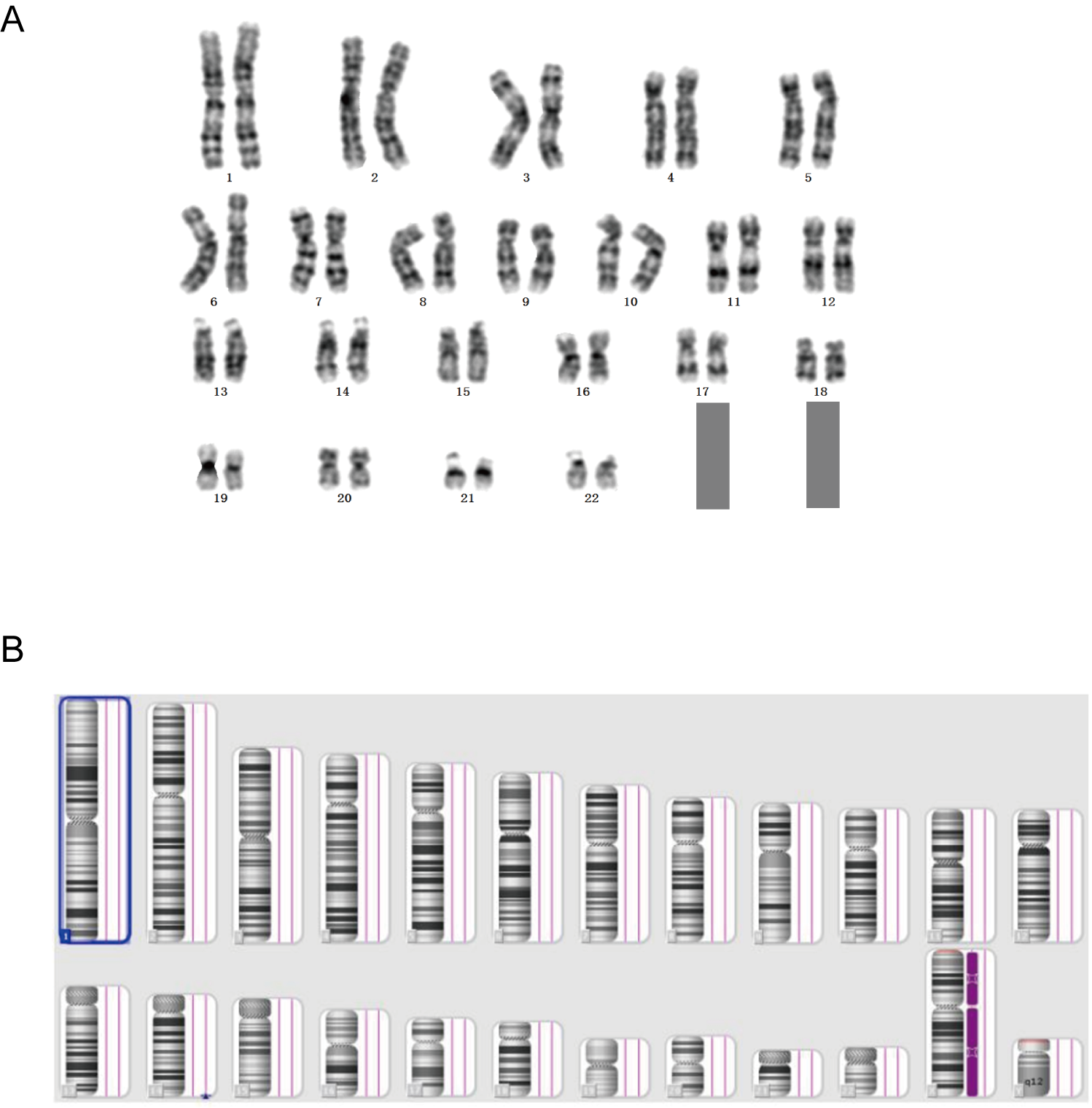

Supplement: Supplementary file 3 [file Image3.tif]
